# Merger of Ayurveda and Tissue Culture-Based Functional Genomics: Inspirations from Systems Biology

**DOI:** 10.1186/1479-5876-6-14

**Published:** 2008-03-18

**Authors:** Custer C Deocaris, Nashi Widodo, Renu Wadhwa, Sunil C Kaul

**Affiliations:** 1 National Institute of Advanced Industrial Science & Technology (AIST), Central 4, 1-1-1 Higashi, Tsukuba, Ibaraki 305-8562, Japan

## Abstract

Ayurveda is one of the ancient systems of health care of Indian origin. Roughly translated into "Knowledge of life", it is based on the use of natural herbs and herb products for therapeutic measures to boost physical, mental, social and spiritual harmony and improve quality of life. Although sheltered with long history and high trust, ayurveda principles have not entered laboratories and only a handful of studies have identified pure components and molecular pathways for its life-enhancing effects. In the post-genomic era, genome-wide functional screenings for targets for diseases is the most recent and practical approach. We illustrate here the merger of ayurveda and functional genomics in a systems biology scenario that reveals the pathway analysis of crude and active components and inspire ayurveda practice for health benefits, disease prevention and therapeutics.

## No longer a crude view of the crude extracts

The word "Ayurveda" comprises of word *āyus *meaning "life" or "life principle", and the word *veda*, which refers to a system of "knowledge". Thus "Ayurveda" roughly translates as the "knowledge of life". Ayurveda is a 5000+ year-old system of Indian home medicine using natural plant extracts. With over 400,000 registered Ayurveda practitioners, this system of medicine remains as one of the most ancient yet living tradition practiced widely in India, Pakistan, Sri Lanka and in other countries. The basic principle in Ayurveda is to give greater priority to total wellness and health rather than to make selective treatment. It has become very popular as it uses reagents and remedies essentially drawn from nature and is both eco- and bio-friendly. Ayurvedic medical system practices the use of dry powder or crude extract, and assignment of bioactivities to a particular compound is not preferred. Interestingly, the mainstream pharmaceutical research is also on its way towards veering from mono-molecular or single target approach to combinations and multiple target strategies [1]. Perhaps, multi-site mechanisms of action of herbal preparations from the crude extracts may offer greater chances for success where conventional single-site agents have been disappointing. Auspiciously, many of these traditional herbal medicines are now increasingly being appreciated with Western models of integrative health sciences and evidence-based approach both in research and practice. Several bioactive compounds have emerged from research in Ayurvedic herbals. Among others include, *Rauwolfia *alkaloids for hypertension, psoralens for vitiligo, *Holarrhena *alkaloids in amoebiasis, guggulsterones from *Commiphora *as hypolipidemic agents, *Mucuna pruriens *for Parkinson's disease, bacosides from *Bacopa monnieri*, antivirals from phyllanthins, withanolides and many other steroidal lactones and their glycosides as immunomodulators [2-4].

Despite the recent revolutions in biotechnology and genome research, an estimated 80% of the world population still has no access to modern medicine and obtain benefits from the time-tested alternative systems of medicine [5]. As more genomes have been sequenced and gene functions elucidated, time has come to bring the valuable ancient medicinal knowledge to the rapidly expanding genomic landscapes. It is envisioned that systematic identification and characterization of gene targets could lead to deeper appreciation of the chemo-diversity in an herbalist's "brown bag". It is, however, feared that by reducing traditional medicine to their mere molecular effectors the investigators could "lose sight of the forest for the trees". And as such, inspired by the holistic character of traditional medicinal systems to study functions of drug-responsive genes, we have entertained the idea of applying a systems biology perspective in comparing gene regulatory circuits in an herbal preparation vis-à-vis some of its bioactive components. A global accounting of molecular pathways depicted by a cellular wiring diagram would offer an appreciation of how the activity of individual component–by influencing each other–ultimately translates into the phenotypic response of a target cell, e.g. tumor. The manuscript presents the concept of systems biology for herbal medicine and the implications of our recent cell-based functional genomic work towards clarifying the anti-cancer properties of one of the most prominent therapeutic plant of Ayurveda, the Ashwagandha (ASH).

## From the active component to crude extract of Ashwagandha (ASH) – back to basics?

ASH (*Withania somnifera)*, also known as winter cherry, Indian ginseng or rasayana, is a member of GRAS (Generally Regarded As Safe) plants and a popular home remedy in the Indian pharmacopoeia. Among the Ayurvedic plants, pharmacological and biochemical studies in ASH have been among the most extensive. Its crude extract is included in commercial formulations prescribed for some musculoskeletal problems (e.g., arthritis and rheumatism), and as a general tonic for overall health and longevity [6]. Despite being under-appreciated in the area of oncology [7], experiments in test tubes and in animal models demonstrated that ASH plays an anticancer role by inducing apoptosis and cell cycle arrest, enhancing the immune system, and inhibition of angiogenesis and metastasis. Interestingly, as ASH exhibits both anti-oxidant and pro-oxidant activities, it has been reported to sensitize tumors to radiation while presenting itself a radio/chemo-protector for normal cells. As this section covers a review of the anti-cancer properties of ASH and its bioactive components, readers will find the recent compendia on its various therapeutic benefits very useful [6-9].

P. Uma Devi, a radiation biologist from Jawaharlal Nehru Cancer Hospital and Research Centre (Manipal, India), was one of the early pioneers to research on ASH effects on cancer growth. When crude alcoholic extract from ASH roots was *i. p*.-injected (200–1000 mg/kg body wt daily for 15 days) in mice, complete regression of injected sarcoma occurred within 100 days [10]. They extended the usefulness of ASH as an anti-cancer agent from a radiation oncology perspective by demonstrating its synergism with other treatment modalities, namely radiotherapy (10 Gy gamma-irradiation) and hyperthermia (43°C for 30 min), a phenomenon that was related to depletion of cellular glutathione with ASH treatment [11]. They demonstrated that the cumulative doses of the extract (500–750 mg/kg daily) did not show any toxicity, in contrast to pure withaferin A that was toxic even at a low dose. The results supported the importance of ASH extract as a novel candidate herbal clinical sensitizer. But before such appreciation of clinical uses of crude extracts came to fore, it is noteworthy that it had taken almost two decades since the first series of reports on the anti-neoplastic potentials of the purified component withaferin A were published [12-15].

ASH has been proposed to regulate the cell cycle and apoptosis pathways in several ways depending on the cell type. First, a methanolic leaf extract of ASH (LASH) was demonstrated to restore normal p53 function in tumor cells bearing mutated copies. Further purification of the tumor-selective inhibitory factor (dubbed as i-Factor) ascribed novel anti-cancer functions to withanone [16,17]. Second, in cases when tumors were p53-null, i.e., HL-60 leukemia cells, the LASH caused apoptosis by down-regulation of bcl-2, cytochrome c release from the mitochondria and caspase-3 activation. After a series of purification, Senthil et al. [18] revealed that the withanolide 5Á-ethoxy-1-oxo-6b, 14a, 17b, 20-tetrahydroxy-20*S*, 22*R*-witha-2, 24-dienolide exhibited an activity comparable to that of the crude extract. Third, the methanolic root extracts, at doses of 65–265 ug/ml, down-regulated expression of p34cdc2, a cell-cycle regulatory protein and could lead to the growth arrest at the G2/M phase. Interestingly, the extract utilized in this study also did not contain withaferin A [19]. Fourth, with molecular simulation withaferin A has been shown to target a threonine residue of proteasomal chymotrypsin and inhibit its chymotrypsin-like activity in human prostate cancer cultures and xenografts. It results in accumulation of ubiquitinated proteins and the three proteasome target proteins (Bax, p27, and IkappaB-alpha) accompanied by androgen receptor protein suppression and apoptosis induction. The ubiquitin proteasome pathway is now widely recognized as an important target for drug discovery, because many important intracellular processes, such as cell cycle progression are orchestrated through the orderly degradation of key regulatory protein factors [19,20]. Fifth, for prostate tumors, withaferin A was able to sensitize androgen receptor (AR)-positive PC-3 prostate tumor cell line to androgen ablation therapy via the prostate apoptosis response-4 (Par-4) gene. This has been initially shown to be independent of p53- and PTEN cascades. Interestingly, while individually, withaferin A and anti-androgens induced neither Par-4 nor apoptosis in the cells, the combination of both synergistically induced Par-4 and apoptosis [21]. Sixth, LASH, but not withaferin A, sensitized human cancer cells to anti-cancer drugs suggesting that the LASH may be employed in combination with anticancer drugs to yield an effective combinatorial anti-cancer formulation [22]. And lastly, the LASH as well as purified withaferin A both inhibited NF-κB activation by preventing the activation of IkappaB kinase beta via a thioalkylation-sensitive redox mechanism. Other steroidal lactones, such as withanolide A and 12-deoxywithastramonolide, showed less inhibitory activity [23]. Ichikawa *et al*. corroborated this finding by showing the structure-function studies on withaferin A and its analogues [24]. The study further emphasized the importance of unsaturated lactone moiety in ring A in withaferin A. Both studies revealed the role of withaferin A in suppressing NF-κB activity through inhibition of IKK leading to the suppression of phosphorylation and degradation of IB. Inhibition of the transcriptional factor NF-κB activation was forwarded as one of the crucial mechanisms in induction of apoptosis, overcoming resistance mechanisms and inhibition of immune response and inflammation mechanisms of ASH.

## Crude extract versus pure compounds of ASH: Results from cell-based assays with functional RNAs

**Figure 1:**
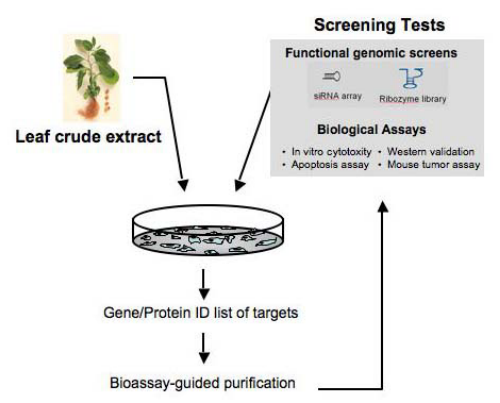
**Diagram of a cell-based genomic-screening for anti-proliferative components of LASH.** Tumor cells were stably transfected either with a siRNA array or a ribozyme library. After exposure to the crude extracts, gene targets were identified. Bioassay-guided purification was done to isolate the active component for the crude extracts. The p53 gene, a convenient target, was among the initial genes identified with LASH.

Our genomic-based screening for the bioactive component in extracts from the leaves of ASH (LASH) that could selectively kill tumor cells (and not normal cells) consisted of two parts (Fig. 1) [16,17]. In the initial phase, tumor-derived and normal cell lines were first treated with crude extract of ASH then their cytotoxicity profiles were studied. As we found an interesting high-degree of selectivity by LASH in exerting its cytotoxic potential in cancer cells, a functional gene screening strategy was then implemented by treating tumor cells with LASH after stable transfections of siRNAs in an array format. We expect that the tumor cells that express either the siRNA or ribozyme targeting a gene essential for the cytotoxic effect of a specific agent should show greater resistance to the particular drug. For the siRNA library, since the sequence and target of the siRNA species is known, we enforced a stringent criterion to avoid false-negatives by choosing the cells that have, at least, two siRNAs aimed at different regions to be LASH-resistant. We found that the p53 pathway was a main target of LASH. More importantly, LASH was able to activate tumor suppressor functions of mutant p53 possibly by inducing allosteric conformation changes based on the gain of wild-type specific epitopes [16].

The second step was a phenotype-guided purification scheme with reverse phase HPLC. By combination of phenotypic effects in cultured normal and cancer cells and gene targets obtained by gene-silencing screenings, we identified the active compounds and the target pathways. To this end we were able to identify (i) withaferin A and i-Factor (withanone) killed cancer cells, (ii) i-Factor caused selective killing of cancer cells and (iii) p53 pathway was selectively altered in cancer cells by i-Factor. Molecular, biochemical and visual analysis also supported that while withaferin A possessed significant toxicities in tumor cells, it also inhibited cell proliferation in normal human embryonic lung (TIG1 and MRC-5) fibroblasts. On the other hand, the activity of (i-Factor) withanone was highly selective only to tumor cells, leaving the normal cells unscathed.

**Figure 2:**
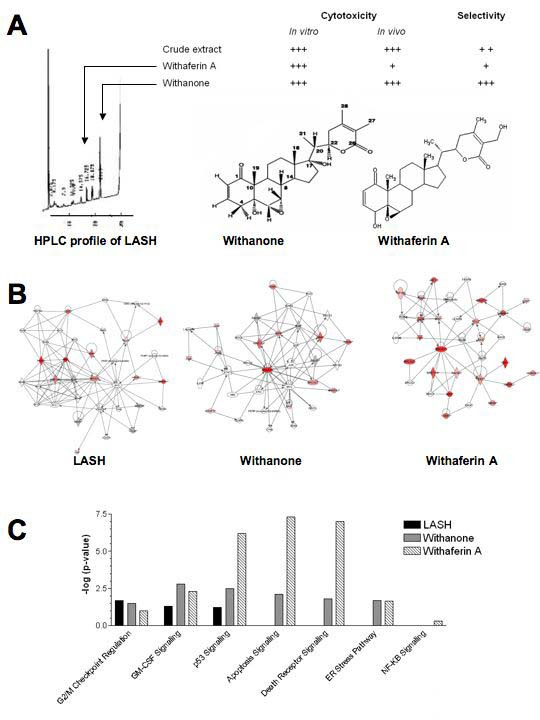
**Molecular networks of crude extracts, withanone and withaferin A.** (A) HPLC fractionation and characterization of the components with anti-tumor activities. LASH crude extract and withanone showed greater selectivity for tumor when assayed in parallel with normal cells. Details of the bioassays conducted are described in Widodo et al. [16]. (B) IPA-generated networks of the withanolides. Shaded *node *indicates that the gene was included in the input list of genes derived from siRNA/ribozyme screening. A *solid line without arrow *indicates protein-protein interaction. *Arrows *indicate the direction of action (either with or without binding) of one gene to another. (C) Pathway comparisons. Fisher's exact test was used at p = 0.05 level to determine whether a biological assignment could be explained by chance alone. Apparent from the canonical pathways, LASH statistically impacted on only three pathways, whereas the effects of withanone and withaferine A were more spread.

As both withanone and LASH gave similar selective cell killing potentials, LASH could be deemed practical for application in cancer management as the crude extract, if proven as efficacious as is easily obtainable compared to the pure compound (Fig. 2A). However, one of the major hurdles in the development of botanical drugs is the lack of scientific acceptance. Some interesting works have emerged in the past few years that identified molecular signatures of a mixture extracted from a medicinal plant extract as opposed to a single component agent [25]. Such insights were made possible through the application of robust gene expression profiling analyses, like the microarray technology or other functional genomic assays, e.g. with siRNAs or ribozymes.

A combined siRNA and ribozyme library screening identified key gene targets for LASH crude extract and its two major anti-tumor components, withanone and withaferin A. We then used the bioinformatics tool "Ingenuity Pathway Analysis (IPA, Mountain View, CA, USA)" to explore how our identified gene targets interact functionally with each other and to gain insights from the differences of the networks that may correlate with the agents' bioactivities. IPA is a commercial, web-based interface that uses a variety of computational algorithms to identify and establish cellular networks that statistically fit the input gene list and/or expression values from experiments. The analysis uses a database of gene interactions culled from literature and updated every quarter of the year.

Given the limited coverage of the siRNA library (enriched for genes involved in apoptosis, cell proliferation and stress response) and the stringency of the ribozyme library screening, gene targets isolated from LASH, withanone and withaferin A were found to be present in the IPA database. The predominant networks for the three agents are shown in Fig. 2B. The top three nodes that act as highly connected hubs are: BRCA1, MYC, and BLM for LASH; BRCA1, MYC and c-JUN for withanone; BRCA1, JAK2 and TERT for withaferin A. Additionally, the cellular networks of the LASH showed greater similarity to withanone (i-Factor) compared to that of withaferin A, and that these interwoven pathways correlated with selectivity in tumor action of the former two agents. It appears from our data that a novel target by withanolides from *W. somnifera *is the BRCA1, a transcription factor implicated in cell cycle regulation, genome integrity, DNA damage response (DDR). Only resveratrol (3,5,4'-trihydroxy-trans-stilbene), a dietary constituent found in grapes and wine, has been found to exert anticancer activity by targeting BRCA1 in breast tumor cell lines [26].

Two studies on genomic comparisons of the crude extract versus its pure bioactive compound have recently been published. Yang et al. [27] performed a comparative pharmacogenomic analysis between *Anoectochilus formosanus *extract, a folk remedy from Taiwan, and its known anti-cancer bioactive component, plumbagin. Using MCF-7 breast cancer cells, they noted that while the pure plumbagin up-regulated 50 known genes and down-regulated 30 others, crude extract that in theory contains a more complicated brew of phyto-compounds, surprisingly affected only half the number of genes at >3-fold difference in expression levels. Subsequent confirmation with Western analysis identified differential oncogenic signaling pathways for the cell killing effects of the two agents: the crude extract up-regulated caspase 8 and cytochrome c and caused apoptosis predominantly while plumbagin resulted in an induction of the cell-cycle checkpoint gene p21 and DNA topoisomerase II leading to cell cycle arrest [27]. The study has provided evidence that the individual components and the crude extract may cause differential signal transductions and the effect of the crude extract may not be the cumulative effect of all of its components. Accordingly, the biological response such as cytostasis or apoptosis may vary depending on the individual components and their composite mixture.

Differences in the gene profiles of the hypothalamus of rats that were administered either by St. John's wort (*Hypericum perforatum*) or imipramine, a synthetic anti-depressant drug, were very intriguing. *H. perforatum *is one of the most popular herbal medicines in Europe and North America, and it is taken by people who suffer from seasonal mood disorders and other types of depression. Hypothalamus was chosen because of its known importance in mood altering functions, and it was suggested that the St. John's wort and imipramine could be expected to share some common pathways leading to their similar beneficial effects. The arrays used by Wong et al. contained 8799 rat genes plus ESTs [28]. To the authors, the results were said to be disappointing since crude extracts of *H. perforatum *differentially regulated a total of only 66 genes, in comparison to imipramine that affected 74 genes. Although variety of different pathways were represented by these genes, the surprising finding was that only six genes were common to the treatments in spite of the similar physiological effects on the animals. In addition to these small numbers, the actual magnitude of the changes was generally small (<2-fold change), although these were claimed to be significant [28]. The study identified the critical signal transduction pathways involved in the biological response and also suggested that the minor changes in gene expression are sufficient to evoke major responses.

It has often been assumed that the whole crude extract is more useful than each of the purified component because of synergistic actions. On the contrary, based on our finding and observations from other investigators, it is interesting to note that the purified components, i.e. withanone and withaferin A, showed greater diversity in affected pathways such as those relevant to apoptosis, death receptor signaling, endoplasmic reticulum (ER) stress and NF-KB signaling, as compared to the crude LASH extract. The most significant canonical pathways associated with the crude extract LASH were cell cycle checkpoint/G2-M transition GM-CSF signaling and p53 signaling (Fig. 2C).

**Figure 3:**
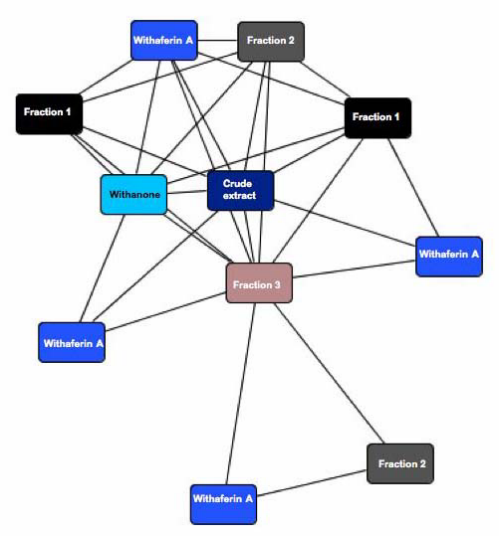
**Global topology of the network interactions among the components of the crude extract.** Each box network identified by IPA based on the input gene list corresponding to the gene target of each HPLC purified component. Note, the centrality of the molecular network affected by the crude extract. This could indicate a synergism of all the components could lead to a convergence to that of the crude extract.

Thus, as it seems paradoxical that the sum of pathways for individual components would, on the contrary, yield to a lesser number compared with the crude extract, a global analysis of the interactome of the five components (wihtanone, withaferin A and three uncharacterized fractions) may provide a rational explanation. As shown in Fig. 3, a simplified representation of network-web showed that the crude extract from LASH occupy the central 'hub' position interconnecting all the pathways representing its individual components. This apparent 'design principle' implicates a special property of the crude extract since the individual components branch-off with robust yet low-connectivity networks, the crude extract maintained the essential few network hub that have the greatest overall effect on cell growth and proliferation. We hypothesize that the interaction between components of the crude extracts may either lead to a synergy of cell killing effects or neutralize the toxicity of each other. This could be significant to the greater appreciation of herbal preparation in mainstream medicine.

## Traditional medicines and inspirations from systems biology: Concluding remarks

Recent developments in computational biology and bioinformatics have provided biologists with some systematic methods to analyze these molecular networks in a cellular context. Collectively predicated as systems biology, it aims to analyze relationships among elements (nodes) in a given system or the emergent properties of the system. Cellular networks that model the cellular response to a given perturbation would include protein-protein interaction networks (PPI: encode the information of proteins and their physical interactions); signal transduction and gene regulatory networks (STN and GRN: show regulatory relationships between transcription factors and/or regulatory RNAs, as well as the signaling pathways that confer these responses); and the metabolic networks (MN: illustrates the biochemical reactions between metabolic substrates and products). Molecular networks that occur in a cell can be presented as either directed or undirected graphs. For example, PPI networks use undirected graphs where nodes represent proteins and the links show the physical interactions between the proteins. An exhaustive description of these networks is available [29].

What insights can we gain by analyzing the web of complexity of these networks associated with herbal drug action in a cell in system biology perspective? We should expect a major contribution from the bio-informatics resources towards the development and enrichment of traditional herbal medicine because such a perspective captures the uniqueness and complexity of drug action in a cell. Such holistic perspective also avoids the pitfall of being too reductionist, and in effect, mollifies some criticisms from traditional ethnopharmacologic researchers.

By going to a reverse direction, our current study likewise tells us that by looking at the entire system of a crude formulation, we can focus on some key central genes, in the case of *W. somnifera*, withanolides is BRCA1, with which the entire summation of network of the individual component may have greater probability for convergence. These targets are most likely vulnerable sites in a cancer cell. Kawamura *et al*. demonstrated the practical implication of working on the 'genomic screening'-guided purification in isolation of non-toxic, active component in Japanese herbal medicine formulation Keishi-bukuryo-gan (KGB) that regulates transcription of hemeoxygenase-1 [30]. However, although these biological phenomena are regarded with assumption of a real cellular mechanism, we should be cautious at the same time that these leads may not be definite and are dependent on the quality of the herbal preparation.
